# Pulmonary Deposition and Elimination of Liposomal Amikacin for Inhalation and Effect on Macrophage Function after Administration in Rats

**DOI:** 10.1128/AAC.00700-16

**Published:** 2016-10-21

**Authors:** Vladimir Malinin, Mary Neville, Gina Eagle, Renu Gupta, Walter R. Perkins

**Affiliations:** Insmed Incorporated, Bridgewater, New Jersey, USA

## Abstract

Pulmonary nontuberculous mycobacterial (PNTM) infections represent a treatment challenge. Liposomal amikacin for inhalation (LAI) is a novel formulation currently in development for the treatment of PNTM infections. The pulmonary deposition and elimination of LAI and its effect on macrophage function were evaluated in a series of preclinical studies in healthy rats. The pulmonary deposition of LAI was evaluated in female rats (*n* = 76) treated with LAI by nebulizer at 10 mg/kg of body weight per day or 90 mg/kg per day for 27 days, followed by dosing of dually labeled LAI (LAI with a lipid label plus an amikacin label) on day 28 with subsequent lung histological and amikacin analyses. In a separate study for assessment of alveolar macrophage function, rats (*n* = 180) received daily treatment with LAI at 90 mg/kg per day or 1.5% saline over three 30-day treatment periods followed by 30-day recovery periods; phagocytic and Saccharomyces cerevisiae (yeast) killing capabilities and inflammatory mediator release were assessed at the end of each period. LAI demonstrated equal dose-dependent deposition across all lung lobes and regions. Lipid and amikacin labels showed diffuse extracellular colocalization, followed by macrophage uptake and gradual amikacin elimination. Macrophages demonstrated accumulation of amikacin during treatment periods and nearly complete elimination during recovery periods. No evidence of an inflammatory response was seen. No differences in microsphere uptake or yeast killing were seen between LAI-treated and control macrophages. Neither LAI-treated nor control macrophages demonstrated constitutive inflammatory mediator release; however, both showed normal mediator release on lipopolysaccharide stimulation. LAI is readily taken up by macrophages in healthy rats without compromising macrophage function.

## INTRODUCTION

Nontuberculous mycobacteria (NTM) are a diverse group of species of the genus Mycobacterium that do not cause tuberculosis or leprosy (thus excluding the members of the Mycobacterium tuberculosis complex, M. leprae, and M. lepromatosis). More than 150 NTM species with a broad range of pathogenicity and virulence have been identified (1, 2). M. avium complex (MAC) is the most common species worldwide; however, the species distribution varies geographically. Other common species are M. fortuitum, M. kansasii, and M. abscessus (2). NTM bacteria causing pulmonary NTM (PNTM) disease may form biofilms and appear to infect human lungs through adherence to compromised respiratory mucosa (1, 2).

The clinical manifestations of PNTM infections include the development of bronchiectasis, cavity formation, bronchial ulceration, and granuloma formation; these pulmonary conditions are associated with widespread peribronchial infiltration by mononuclear and epithelioid cells (3, 4). The management of PNTM infections remains challenging and is constrained by the intracellular localization and the formation of biofilms by NTM, which may limit the ability of antibiotics to achieve effective bactericidal levels in the majority of patients (2, 5, 6). In addition, antibiotic resistance (particularly in cases of M. abscessus infection) (2, 7), as well as poor implementation of guideline-based treatment recommendations, may contribute to inadequate treatment, which may further exacerbate the risk of drug resistance (8).

Liposomal aminoglycosides (amikacin and gentamicin encapsulated inside liposomes) have been shown to be effective against M. avium both *in vitro* and in mice when delivered intravenously (9–12). Liposomal encapsulation has been shown to increase the antimicrobial activity of amikacin against multiple bacterial species (13). Amikacin is particularly effective against numerous NTM species (2). Consequently, intravenous amikacin is recommended for many types of NTM infections (2, 14). However, amikacin has a very narrow therapeutic window. This and the intravenous method of administration limit the potential utility of liposomal amikacin in patients with NTM infections (2). Liposomal amikacin delivered by the pulmonary route provides a local sustained-release reservoir and has been shown to be well tolerated (15, 16).

Here we report the results of a series of preclinical studies of liposomal amikacin for inhalation (LAI; Insmed Incorporated, Bridgewater, NJ), a novel formulation of amikacin currently in development for the treatment of patients with refractory PNTM infections (2, 17, 18). LAI is composed of amikacin encapsulated in liposomes (mean size, 250 to 300 nm). The liposomes are composed of dipalmitoylphosphatidylcholine (DPPC) and cholesterol, both of which are natural constituents of lung surfactant (17, 19). A proprietary efficient manufacturing process was used and provided a high liposomal antibiotic load with an amikacin/lipid mass ratio of ∼1.4 (19). On the basis of the drug/lipid ratio and the liposome size, the within-liposome amikacin concentration is estimated to be 200 to 300 mg/ml. This allows LAI to have a total amikacin concentration of 70 mg/ml, making it possible to deliver therapeutic doses in an acceptable period of time. LAI is delivered by nebulizer, which generates an aerosol of respirable size (mean mass aerodynamic diameter, 3 to 4 μm); this, in turn, enables achievement of high local drug concentrations throughout the distal respiratory airways. More than 98% of the amikacin in LAI is encapsulated within liposomes, and approximately 30% of the encapsulated amikacin is released during nebulization, thus providing immediately available free drug. Pharmacokinetic (PK) data demonstrate that amikacin from deposited LAI is slowly released over time (19, 20). LAI liposomes are neutral in charge and have been shown to readily penetrate mucus and a Pseudomonas aeruginosa bacterial biofilm (17).

In a recent report, *in vitro* treatment of cultured macrophages infected with MAC or M. abscessus with LAI at an amikacin concentration of 10 μg/ml produced significantly more bacterial killing than free amikacin at 10 μg/ml (2). The potential efficacy of LAI against PNTM infections may be attributed to the avid uptake of liposomes by macrophages (2, 21, 22).

A separate study demonstrated that the administration of LAI to healthy rats for 14 days did not produce a significant inflammatory response, nor did it reduce the proportion of macrophages isolated using bronchoalveolar lavage (21). Additionally, macrophages appeared to retain their normal function, measured by determination of both the level of lipopolysaccharide (LPS)-induced production of nitric oxide and tumor necrosis factor alpha (TNF-α) and the level of phagocytosis of fluorescent microspheres (21).

We conducted a series of preclinical studies in healthy rats to characterize the pulmonary deposition and elimination of LAI in the lungs and to further explore the uptake of LAI by alveolar macrophages and the effect of LAI on macrophage functions.

## MATERIALS AND METHODS

### Ethical conduct.

The studies with animals described here were conducted according to the guidelines of the Institutional Animal Care and Use Committee (IACUC) of Insmed Incorporated.

### Pulmonary deposition and elimination of LAI using a dually labeled fluorescent formulation in a 28-day study.

A total of 76 CDIGS female rats (Charles River Laboratories, Inc., Wilmington, MA) were randomized 1:1 into two treatment groups on the basis of the administered dosage (LAI at 10 mg/kg of body weight per day, *n* = 36; LAI at 90 mg/kg per day; *n* = 36) and one control group (1.5% saline, *n* = 4).

LAI was prepared by Insmed Incorporated using a proprietary manufacturing process (19). Dually labeled LAI was prepared by the same process but on a smaller scale as LAI with addition of 1 mol% amikacin-tetramethylrhodamine (TAMRA) (amikacin label) and 1 mol% 1,1′-dioctadecyl-3,3,3′,3′-tetramethylindodicarbocyanine-5,5′-disulfonic acid [DiIC_18_(5)-DS]; it was then administered as a preparation (labeled LAI) containing four parts unlabeled LAI plus one part dually labeled LAI. The construction and testing of the amikacin-TAMRA and DiIC_18_(5)-DS lipid label as probes for amikacin and LAI are described in the Appendix.

LAI and labeled LAI were aerosolized using Pari LC Star nebulizers (Pari, Monterey, CA) and administered to rats using a 12-port Jaeger-NYU nose-only inhalation exposure system (CH Technologies, Westwood, NJ).

LAI (at doses of 10 mg/kg per day or 90 mg/kg per day) was administered to rats in the corresponding treatment groups daily on days 1 through 27. The targeted doses were calculated on the basis of the aerosol concentration in the chamber and the animal minute respiratory volume, according to the Association of Inhalation Toxicologists (AIT) Working Party recommendation for standard delivered dose calculation (23), and were delivered over a single administration of approximately 11 min for the 10-mg/kg dose and over 100 min for the 90-mg/kg dose. On day 28, 12 rats in each treatment group received a single dose of LAI of 90 mg/kg; the remaining 24 rats in each group received a single dose of labeled LAI of 90 mg/kg. This last dose of 90 mg/kg was used to assess the elimination of LAI components from the lung over the consequent extended time period and to ensure a sufficient signal from the bioanalytical tests. The rats were sacrificed over a 28-day period after the last dose (immediately after the dose and at 6 h, 1 day, 3 days, 1 week, and every week after that), and fluid and tissue samples were collected for analysis.

Blood samples were collected from each animal by cardiac puncture at the time of necropsy. Serum was isolated by allowing the blood to clot at room temperature for at least 1 h, and then the blood was separated by centrifugation at 500 × *g* for 10 min. Body and organ weights were recorded at the time of necropsy, and the lungs were separated into individual lobes (cranial, accessory, median, caudal, and left). The left lung was then divided along the transverse plane into approximately three equal sections using a scalpel. The section proximal to the nasal cavity was identified as the top lobe, the section distal to the nasal cavity was identified as the bottom lobe, and the section between the top and bottom lobes was identified as the midlobe.

The individual sections of the left lungs and the cranial, accessory, and median lobes of the right lungs were each homogenized separately in 2 to 5 ml of deionized water per gram of tissue. Lung homogenates were analyzed for DiIC_18_(5)-DS and amikacin-TAMRA concentrations fluorimetrically, whereas total amikacin levels were determined using an immunofluorescence polarization method (with an Abbott TDx automated fluorescence polarization analyzer).

The right caudal lobes of the lungs were frozen and later cut into thin sections (7 μm) using a cryostat microtome. The tissue sections from animals that received nonlabeled LAI were mounted on microscope slides and fixed in solution containing formaldehyde and glutaraldehyde. After fixation, the tissue sections were permeabilized with 0.1% Triton X-100, washed extensively, and then stained with rabbit antiamikacin antibody and a secondary antibody, fluorescently labeled goat anti-rabbit IgG(fab)_2_. Tissues were examined using a fluorescence microscope. Images of tissues from all groups at all time points were photographed and examined for differences in fluorescence intensity. Caudal sections from animals dosed with labeled LAI were mounted on microscope slides without fixation and examined for the deposition of LAI fluorescently labeled with TAMRA and DiIC_18_(5)-DS using a fluorescence microscope.

Amikacin-TAMRA and DiIC_18_(5)-DS concentrations in tissue samples were measured by fluorimetry as described below.

### Effects of LAI on rat alveolar macrophage function in an *in vivo* multiday dosing/recovery cycling study.

A total of 180 CDIGS rats (male rats, *n* = 90; female rats, *n* = 90; Charles River Laboratories, Inc., Wilmington, MA) were randomized into treatment and control groups. The rats in the treatment group (*n* = 108) were further randomized into nine subgroups of 12 rats each (6 male and 6 female rats) and treated with LAI 90 mg/kg per day. The rats in the control group (*n* = 72) were further randomized into six subgroups of 12 rats each (6 male and 6 female rats) and treated with aerosolized 1.5% saline solution. All rats were exposed to three 30-day dosing periods followed by 30-day recovery periods.

Following each 30-day dosing period and 30-day recovery period, 12 rats (6 male and 6 female rats) from each group were euthanized. For each group of 12 rats, lungs from 4 rats (2 male and 2 female rats) were fixed in 10% buffered formalin for histopathologic analysis; lungs from the remaining 8 rats (4 male and 4 female rats) were weighed and lavaged with Hanks' balanced salt solution (HBSS). The resulting bronchoalveolar lavage fluid (BALF) was centrifuged to separate it into cellular and supernatant fractions; the supernatant fractions were stored at −80°C for subsequent cytokine analysis, while the cellular fractions were washed and the cells were counted.

Lung macrophage function was evaluated in three ways. Phagocytic capability was assessed using the uptake of opsonized fluorescent microspheres, while killing capability was assessed using Saccharomyces cerevisiae in a classical assay for determination of the number of CFU. The stimulated release of inflammatory mediators was assessed by culturing macrophages in the presence and absence of lipopolysaccharide (LPS) for 24 h; supernatants were collected by centrifugation, stored at −80°C, and then assayed for the concentrations of nitric oxide (measured as total nitrites) and TNF-α. Details on the methods used are described in the supplemental material.

Amikacin concentrations in lung homogenates and bronchoalveolar lavage fluid were determined by an immunopolarization assay using a Abbott TDx automated fluorescence polarization analyzer as described below.

### Statistical analysis.

The comparisons between two groups were performed using a Student's 2-tailed *t* test. The comparisons among three or more groups were performed using a one-way analysis of variance (ANOVA) model within Prism software (GraphPad). Group differences in mean values were accepted as statistically significant when the corresponding *P* values were less than 0.05.

## RESULTS

### Pulmonary deposition and elimination of amikacin administered as LAI.

**FIG 1:**
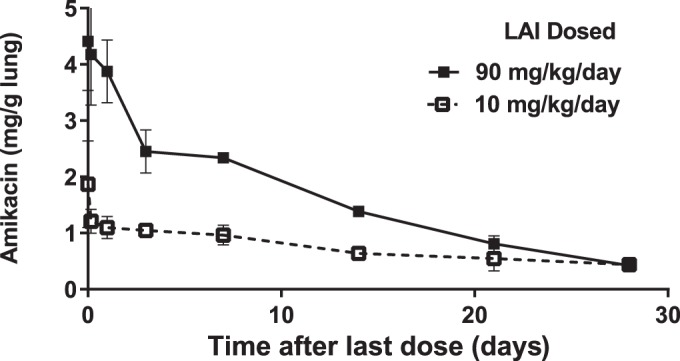
Amikacin deposition and elimination from lungs in rats after 27 days of LAI administration at doses of 10 mg/kg per day and 90 mg/kg per day, followed by a dose of 90 mg/kg on day 28. The symbols and bars (*n* = 3 or 5) represent the means and standard deviations of the amikacin concentration.

Daily treatment of rats with LAI at doses of 10 mg/kg per day and 90 mg/kg per day for 27 days, followed by a single dose of labeled LAI at 90 mg/kg, resulted in the dose-dependent accumulation of amikacin in the lungs, even though the last dose was the same for both groups, as indicated by the levels at time zero in Fig. 1. The elimination of amikacin from the lungs of rats exposed to 90 mg/kg of LAI was notably faster than the elimination from the lungs of rats that were dosed at 10 mg/kg. The average slope of the decline after the initial fast decrease in the first hours was approximately 5 μg/g/h in the high-dose group, whereas it was 1 μg/g/h in low-dose group. As a result, at day 28 after the last dose, amikacin levels became similar regardless of the dosing level.

**FIG 2:**
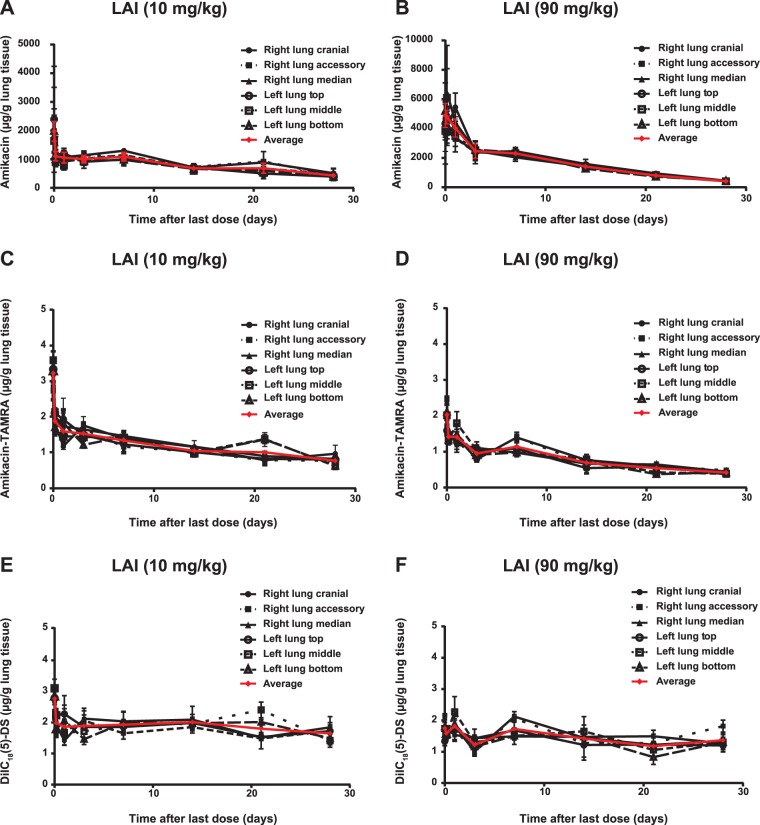
Local and regional deposition and elimination of amikacin (A, B), amikacin-TAMRA (C, D), and DiIC_18_(5)-DS (E, F) in rats after 27 days of treatment with LAI at 10 mg/kg per day or 90 mg/kg per day and labeled LAI at 90 mg/kg on day 28. The symbols and bars (*n* = 3 or 5) represent the means and standard deviations of the concentrations in each lobe.

The deposition and elimination of amikacin LAI were similar across all lung lobes in both the low- and high-dose groups (Fig. 2). The amikacin lung concentration immediately after the last dose (time zero in Fig. 2A and B) was higher in the 90-mg/kg group, consistent with the results presented in Fig. 1. Conversely, labeled LAI deposition was slightly higher in the low-dose group, as indicated by both the amikacin-TAMRA and DiIC_18_(5)-DS lung concentrations at time zero in Fig. 2C to F.

**FIG 3:**
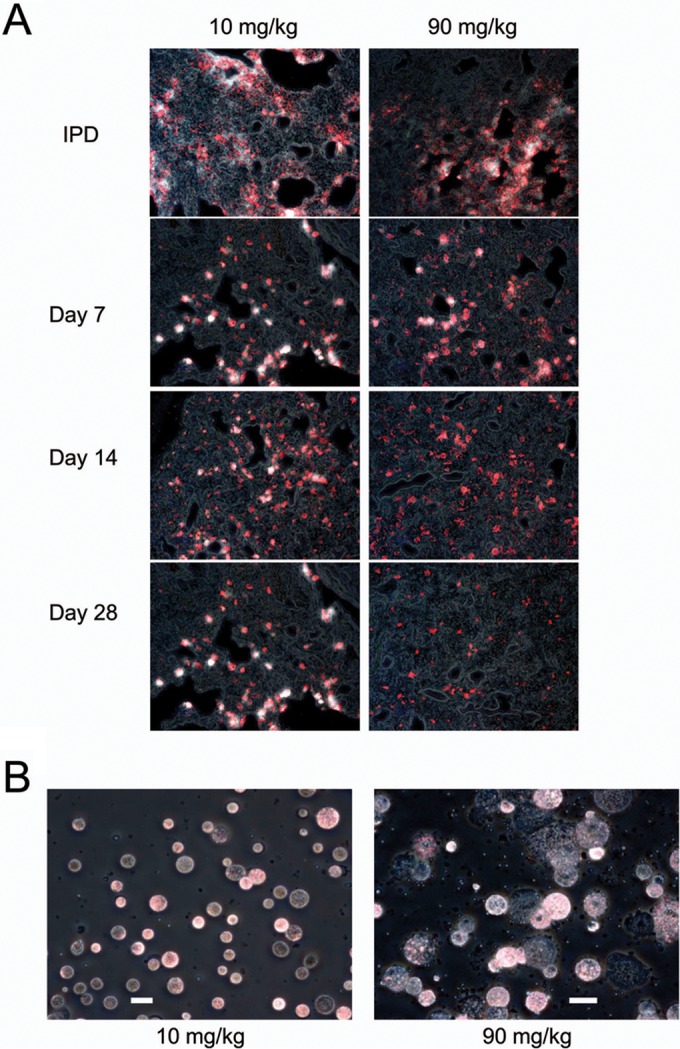
Colocalization of amikacin-TAMRA (white) and DiIC_18_(5)-DS (red) in lung tissue (A) and bronchoalveolar lavage fluid (B) macrophages of rats after 27 days of treatment with LAI at 10 mg/kg per day or 90 mg/kg per day and labeled LAI at 90 mg/kg on day 28. IPD, immediately postdose. Scale bars represent 20 μm.

Within the lungs, amikacin-TAMRA (white) and DiIC_18_(5)-DS (red) fluorescence was primarily colocalized, especially at later times after treatment, indicated as pink areas (Fig. 3). During the first 24 h after treatment, a significant portion of amikacin-TAMRA appeared to be diffuse and extracellular, consistent with LAI releasing 30% of the amikacin from liposomes during nebulization. The majority of that liberated amikacin (and amikacin-TAMRA) was eliminated from the lung within the first 24 h, as indicated by the fast decline in Fig. 2C. The remaining fraction of amikacin stayed within the liposome and thus appeared to be colocalized with lipids, represented as DiIC_18_(5)-DS fluorescence. From 24 h through day 28 after treatment, the liposomal fraction appeared to be taken up by macrophages in the interstitium, bronchi, and alveoli, followed by the gradual release and elimination of amikacin. These patterns of colocalization were observed for amikacin-TAMRA and DiIC_18_(5)-DS fluorescence in lung tissues until 28 days after treatment in both dose groups (Fig. 3A).

Macrophages found in BALF also demonstrated the colocalization of amikacin-TAMRA and DiIC_18_(5)-DS fluorescence; note that macrophages exposed to LAI at 90 mg/kg per day were significantly larger than those exposed to LAI at 10 mg/kg per day, suggesting that more liposomes were taken up at the higher dosage during the 27 days of dosing with nonlabeled LAI (Fig. 3B).

### LAI effects on macrophage morphology and function.

**FIG 4:**
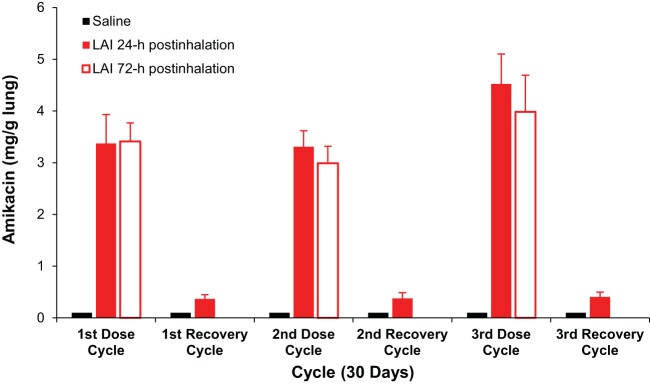
Deposition and elimination of amikacin from lungs of rats following 30-day cycles of treatment with LAI at 90 mg/kg per day and during each 30-day recovery period. The columns and bars represent the means and standard deviations of the concentrations of amikacin in 8 lung homogenates in each group. The mean concentrations of amikacin in all groups of rats treated with 1.5% saline were below the lower limit of quantitation. There were no significant differences in the mean concentrations of amikacin in the lungs of rats that received LAI between lungs harvested at 24 h postinhalation and lungs harvested at 72 h postinhalation in any cycle. There were significant differences (*P* < 0.05) in the mean concentrations of amikacin in the lungs of rats that inhaled LAI between the first and third cycles and the second and third cycles but not between the first and second cycles. Statistical comparison was performed by *t* test using GraphPad Prism software.

Treatment of rats with LAI at 90 mg/kg per day for 30 days resulted in the deposition of more than 3 mg amikacin per gram of lung tissue during each treatment cycle; however, approximately 90% of the amikacin was cleared from the lungs (to levels of <0.5 mg/g of lung tissue) during each subsequent 30-day recovery period (Fig. 4).

**FIG 5:**
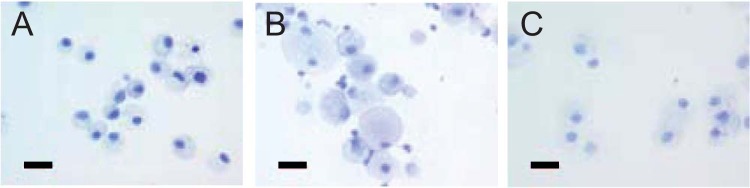
Changes in alveolar macrophage morphology in rats after treatment with 1.5% saline (A) or liposomal amikacin for inhalation at 90 mg/kg per day (B) and after a 30-day recovery period (C). Scale bars represent 20 μm.

Histologic evaluation of lung tissue following LAI administration revealed a diffuse infiltration of foamy macrophages in the alveolar spaces with no evidence of an acute or delayed inflammatory response; no such changes were noted in rats treated with 1.5% saline (Fig. 5A and B). However, following each 30-day recovery period after LAI treatment, the number of alveolar macrophages declined sharply, and their morphology was similar to that observed prior to treatment (Fig. 5C).

**FIG 6:**
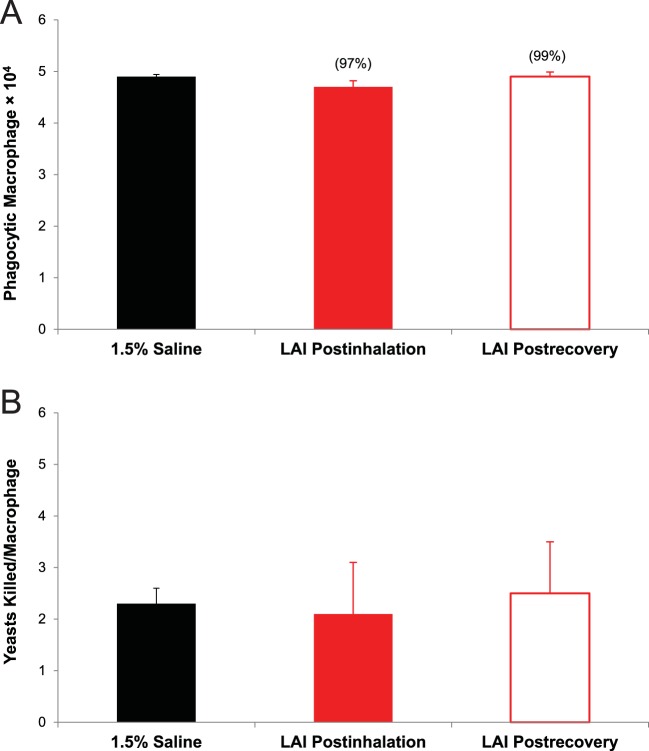
Macrophage phagocytic function (A) and macrophage yeast-killing function (B) in rats after 30 days of treatment with 1.5% saline or LAI at 90 mg/kg per day and after a 30-day recovery period. The columns and bars represent the means and standard deviations of the results obtained for 8 rats in each group. There was no significant difference (*P* > 0.05) in either the number of viable phagocytic macrophages or the number of yeasts killed per macrophage between rats that inhaled 1.5% saline and rats from the postinhalation groups and the recovery groups when the difference was analyzed by *t* test. No significant difference was found between the LAI-treated rats from both the 24-h postinhalation groups and the 72-h postinhalation groups.

Compared with the findings for control rats receiving 1.5% saline treatment, rats receiving LAI demonstrated normal phagocytosis of opsonized fluorescent microspheres following 30 days of treatment, as well as after the subsequent 30-day recovery period (Fig. 6A). Similarly, no effect on macrophage Saccharomyces cerevisiae (yeast) killing capability was observed following 30 days of control or LAI treatment or after the subsequent 30-day recovery period (Fig. 6B).

**FIG 7:**
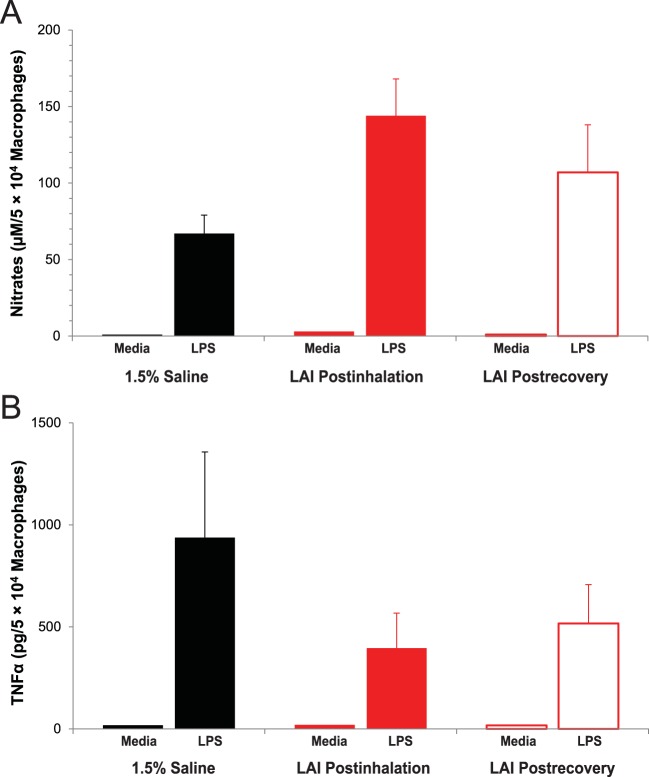
Constitutive and LPS-induced release of the inflammatory mediators nitric oxide (A) and TNF-α (B) by cultured macrophages from bronchoalveolar lavage fluid of rats after 30 days of treatment with 1.5% saline or LAI at 90 mg/kg per day (LAI Postinhalation) and after a 30-day recovery period (LAI Postrecovery). The columns and bars represent the means and standard deviations of the results obtained for 8 rats in each group. A significant increase in the level of production of nitrites by LPS-stimulated macrophages from the LAI-treated rats (in both postinhalation and postrecovery groups) compared with those from 1.5% saline-treated rats was observed (*P* < 0.0001 and *P* < 0.03, respectively). There was also a significant decrease (*P* < 0.001) in the level of production of nitric oxide by macrophages from the LAI postrecovery group compared with the level of production of nitric oxide by macrophages from the LAI postinhalation group. A significant decrease in the level of production of TNF-α by LPS-stimulated macrophages from the LAI-treated rats (in both postinhalation and postrecovery groups) compared with those from the 1.5% saline-treated rats was observed (*P* < 0.001).

In addition, no evidence of the constitutive release of the inflammatory mediators nitric oxide or TNF-α after culturing of macrophages isolated from BALF for 24 h following 30 days of 1.5% saline treatment, LAI treatment, or recovery after LAI treatment was seen (Fig. 7). However, BALF-isolated macrophages retained the ability to release significant concentrations of both nitric oxide and TNF-α when cultured in the presence of LPS, regardless of the treatment.

## DISCUSSION

The studies described herein demonstrated that LAI administered by nebulizer penetrates deeply and is distributed throughout all lobes of the lungs of rats, including the caudal lobe. LAI achieved pulmonary concentrations (up to 4 mg/g of lung tissue at an LAI dose of 90 mg/kg per day; Fig. 1 and 4) that were well above the typical MICs (up to 32 μg/ml for most isolates) for organisms implicated in NTM infections (24, 25).

These studies confirmed that macrophages act as a depot for LAI, with substantial dose-dependent uptake followed by the gradual release and elimination of amikacin. Following LAI uptake by macrophages, amikacin remained closely associated with the labeled lipid (liposomal) fraction, suggesting either that liposomes remain intact or that both lipid and amikacin are retained in equal proportions for a prolonged period. Uptake of LAI by macrophages with repeat dosing can lead to the accumulation of LAI inside the cells, potentially affecting their function. However, the results presented here show that amikacin release and elimination rates appear to be unaffected after repeat dosing at both 10 mg/kg per day and 90 mg/kg per day. Furthermore, the ability to phagocytize microspheres and to affect yeast killing remained intact, and while no evidence of the constitutive release of the inflammatory mediators nitric oxide and TNF-α was seen, macrophages retained the ability to respond to LPS with the release of those mediators.

The design of the studies (i.e., animals were dosed at two levels, 10 mg/kg per day and 90 mg/kg per day for 27 days, with the last dose of 90 mg/kg for all groups being given on day 28) allowed us to assess the effect of subchronic exposure to LAI at low and high doses on lung deposition and elimination after the subsequent administration of labeled LAI at a testing dose. This last, testing dose was the same as the high dose in the previous 27 days (i.e., 90 mg/kg). This design complicates the direct comparison of amikacin deposition, as there appeared to be accumulation of amikacin from the prior 27 days that carried over into the last dose and added to the value of the maximum amikacin concentration in the lung measured immediately after the testing dose at day 28. The concentration of amikacin in the lungs after the last dose (time zero in Fig. 1) was ∼3-fold higher in the high-dose group than the low-dose group. One can estimate the contribution of the 27-day accumulation to the final level to be about ∼75% for the 90-mg/kg dose group and ∼25% for the 10-mg/kg dose group, assuming dose-proportional deposition in a single administration.

However, when looking at labeled LAI deposition and elimination, this preaccumulation of amikacin did not affect the measurement of amikacin-TAMRA and DiIC_18_(5)-DS, as they were administered on day 28 only. Unlike the amikacin deposition shown in Fig. 1, the level of labeled LAI deposition was slightly higher in the low-dose group [amikacin-TAMRA and DiIC_18_(5)-DS at time zero in Fig. 2C to F] but quickly approached a level similar to that in the high-dose group within 1 day. These differences in LAI deposition and elimination can be attributed to differences in macrophage recruitment and intracellular liposome loading, among others. Nonetheless, the rate of amikacin-TAMRA elimination in the high-dose group was not negatively affected compared with that in the low-dose group and was, in fact, faster, similar to the elimination of amikacin.

Taken together, the results of the current study indicate that LAI, after multiple administrations by inhalation, provides a long-lasting elevated local concentration of amikacin in the lung, potentially extending therapeutic levels beyond the treatment period. These findings lend further mechanistic support to the potential of LAI in treating PNTM infections.

A previously reported rat study of LAI demonstrated that the retention time of amikacin in the lungs was much longer when it was administered as LAI than when it was administered as amikacin or tobramycin solutions (17). The study also showed that in rats infected with P. aeruginosa, LAI reduced the bacterial load by more than 2 orders of magnitude compared with that achieved with an equal dose of inhaled free amikacin and every-other-day administration of LAI resulted in reductions in the bacterial load to levels comparable to those achieved with twice-daily tobramycin administration (17).

In a mouse model of pulmonary NTM infection, 28 days of inhaled LAI treatment at 76 mg/kg per day significantly reduced the burden of Mycobacterium avium subsp. *hominissuis* compared with the reduction achieved with inhaled saline, and the average CFU counts were lower than those obtained after treatment with intraperitoneal amikacin at 100 mg/kg per day, despite the delivery of a smaller total dose (2). The *in vivo* effectiveness of LAI was comparable to that of injected free amikacin, even though a smaller dose of LAI was delivered. Although there was not a statistically significant difference in the reduction in the number of CFU between the free amikacin group and two of the three LAI treatment groups (the groups receiving a 1-h inhalation every day for 28 days and a 2-h inhalation every other day for 28 days), the mean number of CFU recovered from both of these two experimental groups was smaller than that recovered from the group treated with free amikacin, even though they received 32% less drug. The data suggest that LAI might be more effective than free amikacin in reducing the M. avium subsp. *hominissuis* burden, which concurs with the observation *in vitro*.

The effect of LAI was maintained regardless of whether the same cumulative dose was administered over 1 h daily for 28 days, 2 h daily for 14 days (followed by 14 no-treatment days), or 2 h daily every other day, and there was no evidence of the development of amikacin resistance in lung isolates at 28 days (2).

Prior studies have reported on the effectiveness of LAI in managing PNTM infections. The *in vivo* efficacy of LAI was recently demonstrated in a murine model study of LAI in PNTM infection (2). Rose et al. (2) showed that the liposomal delivery of amikacin is a viable approach for the treatment of intracellular infections and may overcome the poor penetration of cell membranes encountered with aminoglycoside antibiotics. Results presented here demonstrate that macrophages accumulate amikacin during treatment periods due to the active uptake of LAI. The high local amikacin concentrations achieved with LAI administration may provide the additional benefit of reducing the likelihood of developing resistance.

Recent phase 2 clinical studies provided evidence that LAI is well tolerated and provides a sustained effect, allowing once-daily administration. Phase 2 clinical studies of LAI have reported on its preliminary safety and efficacy against P. aeruginosa in patients with cystic fibrosis (CF) (26) and against refractory MAC and M. abscessus lung diseases in patients with CF and non-CF bronchiectasis (27, 28). Currently, a phase 3 study in patients with MAC lung disease who failed prior treatment is under way. Further investigation is warranted.

The avid uptake of LAI by macrophages, along with the retention of normal macrophage functions, may be especially important in the context of effective management of PNTM infections, in which macrophages act not only as the primary line of defense but also as a reservoir for NTM (14, 18, 22). The results of the current studies, along with the results from previous preclinical and clinical studies, support the need for the continued evaluation of LAI in the treatment of PNTM infections.

### Conclusions.

When administered to rats by nebulization, LAI demonstrated dose-dependent accumulation and elimination, with even deposition across all lung regions. LAI was avidly taken up by lung macrophages in a dose-dependent manner, with the consequent extended release of amikacin from the macrophage depot. LAI was not associated with an altered inflammatory response or with macrophage dysfunction. LAI should be further evaluated as a potentially useful therapeutic option in the treatment of PNTM infections.

## APPENDIX

### Development of fluorescent lipid label as a probe for liposomes.

The fluorescent lipid label was developed to satisfy three conditions: a fluorescence emission wavelength that would not interfere with the emission wavelengths of other fluorescent probes (e.g., amikacin-TAMRA), an amikacin leak profile that does not interfere with the leak profile of amikacin from the liposomes, and the ability to create liposomes in which the label was isolated to the inner monolayer, leaving the outer monolayer free to interact normally with the local environment.

The fluorophores *N*-(7-nitrobenz-2-oxa-1,3-diazol-4-yl)-dioleoylphosphatidylserine (NBD-PS) and 1,1′-dioctadecyl-3,3,3′,3′-tetramethylindodicarbocyanine-5,5′-disulfonic acid [DiIC_18_(5)-DS] were considered potential probes: both could be reduced using dithionite to remove the fluorescence associated with the outer monolayer, and both demonstrated a slow flip-flop rate (translocation from the inner to the outer membrane), ensuring stability of the inner-monolayer-only label. DiIC_18_(5)-DS was ultimately selected over NBD-PS as the best candidate for use, primarily because of its higher maximum emission wavelength (670 nm versus 534 nm for NBD-PS); the two probes could be similarly reduced to a nonfluorescent derivative with dithionite treatment of the outside liposome surface, and they showed comparable flip-flop rates that were relatively unaffected by nebulization.

When incorporated into liposomes at a concentration of 1 mol%, DiIC_18_(5)-DS was found to have a minimal effect on the liposome surface charge (∼0.1 mV) that was relatively unaffected by nebulization. In addition, the physical characteristics of the labeled liposomes were similar to those of LAI.

During subsequent testing, it was shown that the rate of detergent-induced leakage was similar between LAI and labeled liposomes both pre- and postnebulization. In addition, fluorescence analysis showed that the addition of DiIC_18_(5)-DS at 1 mol% did not interfere with the detection or quantitation of labeled amikacin.

On the basis of these evaluations, it was concluded that liposomes incorporating DiIC_18_(5)-DS at 1 mol% have the same characteristics as LAI and represent a valid probe for LAI behavior *in vivo*.

### Development of fluorescent amikacin probe.

The fluorescent amikacin probe needed to demonstrate three basic properties: formulation stability, leak profiles (spontaneous and detergent induced) from LAI similar to those from unlabeled amikacin, and patterns of lung elimination similar to those of amikacin. Two candidate fluorophores were considered: tetramethylrhodamine (TAMRA) and Texas Red (TR) with an attached spacer and succinimidyl group. Both were conjugated to amikacin by their succinimidyl ester groups by the formation of a stable amide bond. Ultimately, amikacin-TAMRA was selected as the lead candidate; it demonstrated a peak emission wavelength of 580 nm distinct from that of DiIC_18_(5)-DS and from the background fluorescence of biological fluids.

Amikacin-TAMRA and amikacin showed similar rates of leakage from liposomes when incubated with detergent (rhamnolipid) both before and after nebulization; these rates were unaffected by the incorporation of DiIC_18_(5)-DS at 0.5 mol% into the test liposomes. In addition, the elimination rate of amikacin-TAMRA from lungs, serum, and urine was demonstrated to be similar to that of free amikacin and markedly different from (i.e., slower than) that of free TAMRA.

As part of the pulmonary deposition study described in the main text, the ratios of the area under the concentration-time curve (AUC) and the maximum concentration for amikacin-TAMRA, free amikacin, and free TAMRA were calculated on the basis of immunopolarization and fluorescence.

The results, shown in Table A1, demonstrate that immunopolarization and fluorescence detection of amikacin-TAMRA resulted in similar values. Moreover, the ratios for amikacin-TAMRA and free amikacin were similar and different from those for free TAMRA, demonstrating that the pharmacokinetic behavior of amikacin-TAMRA is similar to that of free amikacin.

**TABLE A1:** Ratios of area under the concentration-time curve and maximum concentration for amikacin-TAMRA, free amikacin, and free TAMRA

Specimen	Avg (range) AUC-to-*C*_max_ ratio^*a*^			
	Amikacin-TAMRA		Free TAMRA fluorescence	Free amikacin immunopolarization
	Immunopolarization	Fluorescence		
Lung	2.7 (2.1–3.2)	2.7 (2.5–2.9)	1.5 (1.3–1.8)	2.4 (2.2–2.5)
Serum	2.3 (2.0–2.6)	2.1 (1.6–2.6)	1.4 (1.3–1.4)	2.6 (1.6–3.6)
Urine	2.6 (1.8–3.1)	2.8 (1.6–3.0)	2.7 (2.6–2.9)	3.5 (3.5–3.6)

a The area under the concentration-time curve (AUC) and the maximum concentration (*C*_max_) were determined graphically by Prism software (GraphPad) for amikacin and amikacin-tetramethylrhodamine (TAMRA) for each of the specimens in triplicate. Ratios were obtained by dividing the AUC of each specimen by its maximum concentration.

## Supplementary Material

Supplemental material
