# Supplementary material for: Pulmonary Deposition and Elimination of Liposomal Amikacin for Inhalation and Effect on Macrophage Function after Administration in Rats
Source: Antimicrob Agents Chemother. 2016 Oct 21;60(11):6540–9. doi: 10.1128/AAC.00700-16 (PMC5075057; doi:10.1128/AAC.00700-16)
Supplement: Supplemental material [file supp_60_11_6540__index.html]

Supplemental material 

# Pulmonary Deposition and Elimination of Liposomal Amikacin for Inhalation and Effect on Macrophage Function after Administration in Rats

## Supplemental material

- Supplemental file 1 -

  Supplemental text and Fig. S1-S3

  PDF, 1.6M
