# Supplementary material for: Pulmonary Deposition and Elimination of Liposomal Amikacin for Inhalation and Effect on Macrophage Function after Administration in Rats
Source: Antimicrob Agents Chemother. 2016 Oct 21;60(11):6540–9. doi: 10.1128/AAC.00700-16 (PMC5075057; doi:10.1128/AAC.00700-16)

MATERIALS AND METHODS

Inhalation Therapy

Aerosols of liposomal amikacin for inhalation (LAI) and 1.5% saline were administered to the rats using 12-port nose-only inhalation chambers (12-port Jaeger-NYU Nose-only Inhalation Exposure System, CH Technologies, Westwood, NJ, USA), air compressors (Model 8650D, DeVilbiss, Concord, Ontario, Canada), and nebulizers, (PARI, Monterey, CA, USA). Concentrations of aerosolized amikacin for each of the CH Technologies nose-only chambers were determined in order to calculate the time required to dose rats with amikacin.

BALF Markers of Inflammation

The bronchoalveolar lavage fluid (BALF) supernatants and supernatants from cultured BALF cells were assayed for tumor necrosis factor-alpha (TNFα) and nitric oxide using commercially available kits (rat TNFα Quantikine ELISA Kit, RTA00, R&D Systems, Minneapolis, MN, USA; and Nitrate/ Nitrite Colorimetric Assay Kit, Cat # 780001, Cayman Chemical, Ann Arbor, MI, USA). The concentrations of urea in the serum and BALF were measured using a QuantiChrom™ Urea Assay Kit (GENDIUR500; Accurate Chemical & Scientific Corporation, Westbury, NY).

Phagocytosis of Fluorescent Microspheres

Macrophages (5 x 10^4) were incubated with opsonized fluorescent microspheres (Fluoresbrite™ carboxy NYO, 0.2 micron microspheres, ~3 x 10^{10} particles/mL, Polysciences, Inc; Warrington, PA) for 1 hr at 37°C in 5% carbon dioxide (CO_2). Wet mounts of MØ diluted 1:1 with trypan blue were prepared. The total number of viable macrophages and of fluorescent macrophages was counted microscopically (Nikon, 500 X magnification). At least 50 macrophages per slide were counted. The percentage of phagocytic macrophages and number of phagocytic macrophages per well were calculated.

Phagocytosis and Killing of *Saccharomyces cerevisiae*

Macrophages (5 x 10^4) were mixed with *S cerevisiae* at a ratio of 1 to 5, respectively, and controls of only yeasts (2.5 x 10^5) were run in triplicate. After 16 to 18 hr at 37°C, supernatants were removed after centrifugation. Macrophages were lyzed with 200 µL of sterile water containing sterile 1% Triton-X100. Lysates (100 µL) were further diluted in tubes with a broth containing yeast extract, peptone, dextrose, and tryptophan (YPD). Fifty (50) µL of these dilutions were spread over YPD agar plates as well as dilutions of the control yeasts which were treated in a similar manner as macrophages. The YPD agar plates were incubated at 30°C for 48 hr. The numbers of colony forming units (CFU) were counted 48 hr after plating. The % yeast killed by MØ and number of yeast killed per macrophage were calculated.

Determination of Amikacin Concentrations in Lung Tissue and in Blood Serum

All rats were euthanized by carbon dioxide asphyxiation, and blood was collected by cardiac puncture of the left ventricle. Selected organs were removed and weighed and samples of biological fluids were collected. Specific lung lobes and lung sections as identified by the protocol were placed into individual 50-mL conical tubes, mixed with 1.5% saline to a total volume of 10 mL, and homogenized with a Polytron® Homogenizer (Brinkmann Instruments, Rexdale, Ontario, Canada) at the maximum speed setting until a smooth homogenate was obtained. A 0.20-mL aliquot of each lung homogenate was diluted with a mixture of TDx™ buffer (Abbott Laboratories, Abbott Park, IL, USA) and 0.5% Triton X-100 at a volume ratio of 1:5 and 1:2 for rats exposed to liposomal amikacin and amikacin, respectively. The resulting suspensions were incubated for at least 10 minutes at room temperature and then clarified by centrifugation (Fisher Scientific, Waltham, MA, USA, Model 235C centrifuge, 5 min at room temperature). An aliquot of each supernatant was then analyzed for the amikacin content.

Sera were normally assayed for amikacin content without dilution. If the concentration of antibiotic in the sera was found to be out of the linear range of the assay, the samples were diluted with TDx buffer and re-assayed.

The amikacin concentration of all lung homogenates and biological fluids was measured by immunopolarization using the Abbott TDx automated fluorescence polarization analyzer (Abbott Laboratories, Abbott Park, IL) according to the manufacturer's instructions. The lower limit of quantification (LLOQ) in the serum samples was approximately 3 µg/mL, whereas the LLOQ for lung homogenates was approximately 25 µg per gram of lung tissue.

Fluorometric Method to Measure the Concentration of DiIC$_{18}$(5)DS and Amikacin-Tetramethylrhodamine (TAMRA) in Rat Lung Homogenate and Blood Serum

Each right lung lobe or left lung section was homogenized in 2.0 mL, 5.0 mL, or 10 mL of distilled water depending on the study design. An aliquot of each lung homogenate (250 µL or 500 µL) was added to 4 mL of n-Propanol/water 50% with 10 mM Tris pH 7 and 1 mM amikacin sulfate in order to dissolve both lipids and amikacin-TAMRA. Amikacin sulfate was included in the solution to help release amikacin-TAMRA from the tissue by competitive binding to possible anionic sites. In addition, amikacin sulfate caused proteins to aggregate and precipitate out of solution, thus reducing background absorbance and background fluorescence of the samples. The resulting mixture was incubated for 10 minutes at 40 °C, cooled to room temperature, and clarified by centrifugation for 10 minutes at 3000 x g. Approximately 3.0 mL of each supernatant was transferred to an optical cuvette and the fluorescence intensity measured using a PTI Fluorometer QM1 (Photon Technology International, Edison, NJ). Blood serum samples were treated in a similar way, except that 100 µL serum aliquot was used.

Amikacin-TAMRA fluorescence was measured at excitation/emission wavelengths of 550/580 nm whereas 1,1′-dioctadecyl-3,3,3′,3′-tetramethylindodicarbocyanine-5,5′-disulfonic acid (DiIC$_{18}$(5)DS) fluorescence was

measured at 650/673 nm. The method was tested for linearity using separate amikacin-TAMRA and $DiIC_{18}(5)DS$ standards in corresponding matrix. Blank tissue matrix was used to determine background fluorescence, which was subtracted from the fluorescence signal.

The Ovalene fluorescent standard (polycyclic aromatic hydrocarbon in a glass block) provided by PTI was used as an internal standard to account for variations in fluorometer sensitivity. Ovalene fluorescence at excitation 422 nm and emission 505 nm was normally at approximately 450,000 cps (counts per second). To quantify each fluorescent probe, the Response Factor was determined by measuring the fluorescence of the probe standards at known concentrations. The ratio of Response Factor to the Ovalene signal was used as a universal factor independent of fluorometer sensitivity.

Figure S1-1. Fluorescence of amikacin-TAMRA (white) and DiIC$_{18}$(5)DS (red) and their co-localization (pink) in lung tissues after 27 daily doses of 90 mg/kg (left) or 10 mg/kg (right) of LAI followed by a single dose of 90 mg/kg of dual-labeled LAI (immediately post dosing, days 1 and 3). DiIC$_{18}$(5)-DS 1′-Dioctadecyl-3,3,3′,3′-tetramethylindodicarbocyanine-5,5′-disulfonic acid; LAI, liposomal amikacin for inhalation; TAMRA, tetramethylrhodamine.

90 mg /kg | 10 mg/kg

Immediately post dosing (IPD)

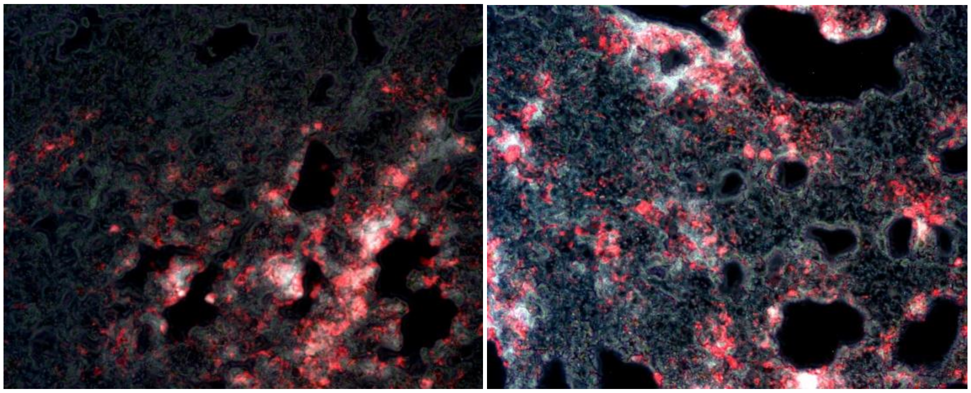

Day 1

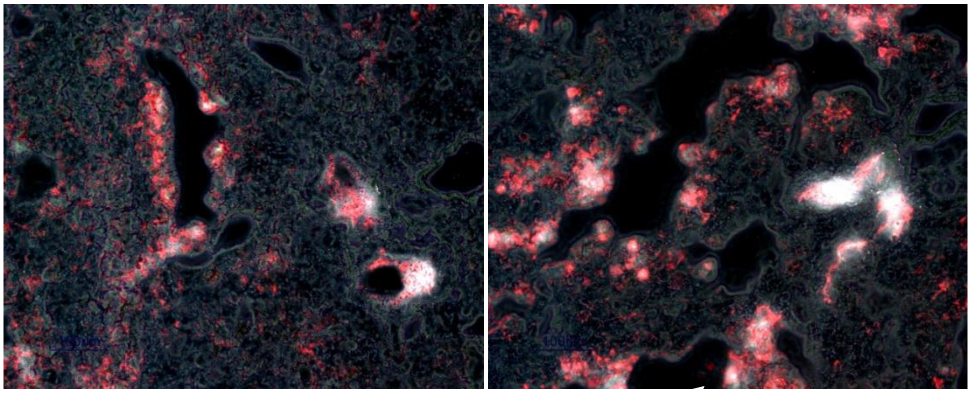

Day 3

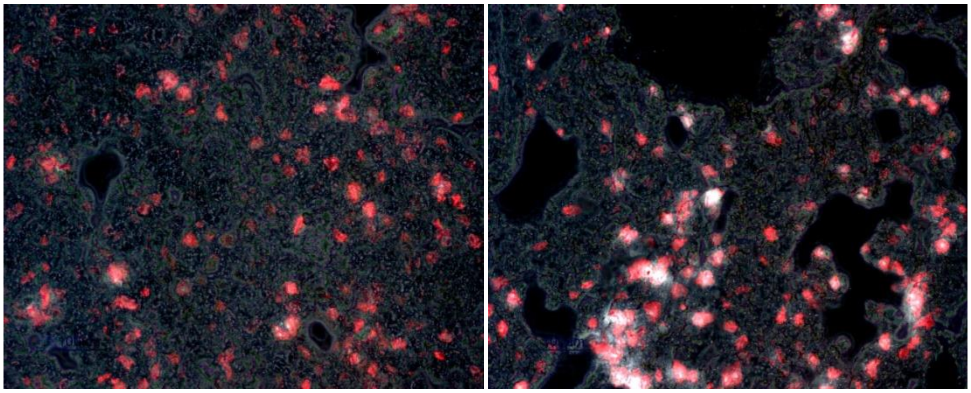

Figure S1-2 . Fluorescence of amikacin-TAMRA (white) and DiIC$_{18}$(5)DS (red) and their co-localization (pink) in lung tissues after 27 daily doses of 90 mg/kg (left) or 10 mg/kg (right) of LAI followed by a single dose of 90 mg/kg of dual-labeled LAI *(days 7, 14, and 21)*. DiIC$_{18}$(5)-DS 1′-Dioctadecyl-3,3,3′,3′-tetramethylindodicarbocyanine-5,5′-disulfonic acid; LAI, liposomal amikacin for inhalation; TAMRA, tetramethylrhodamine.

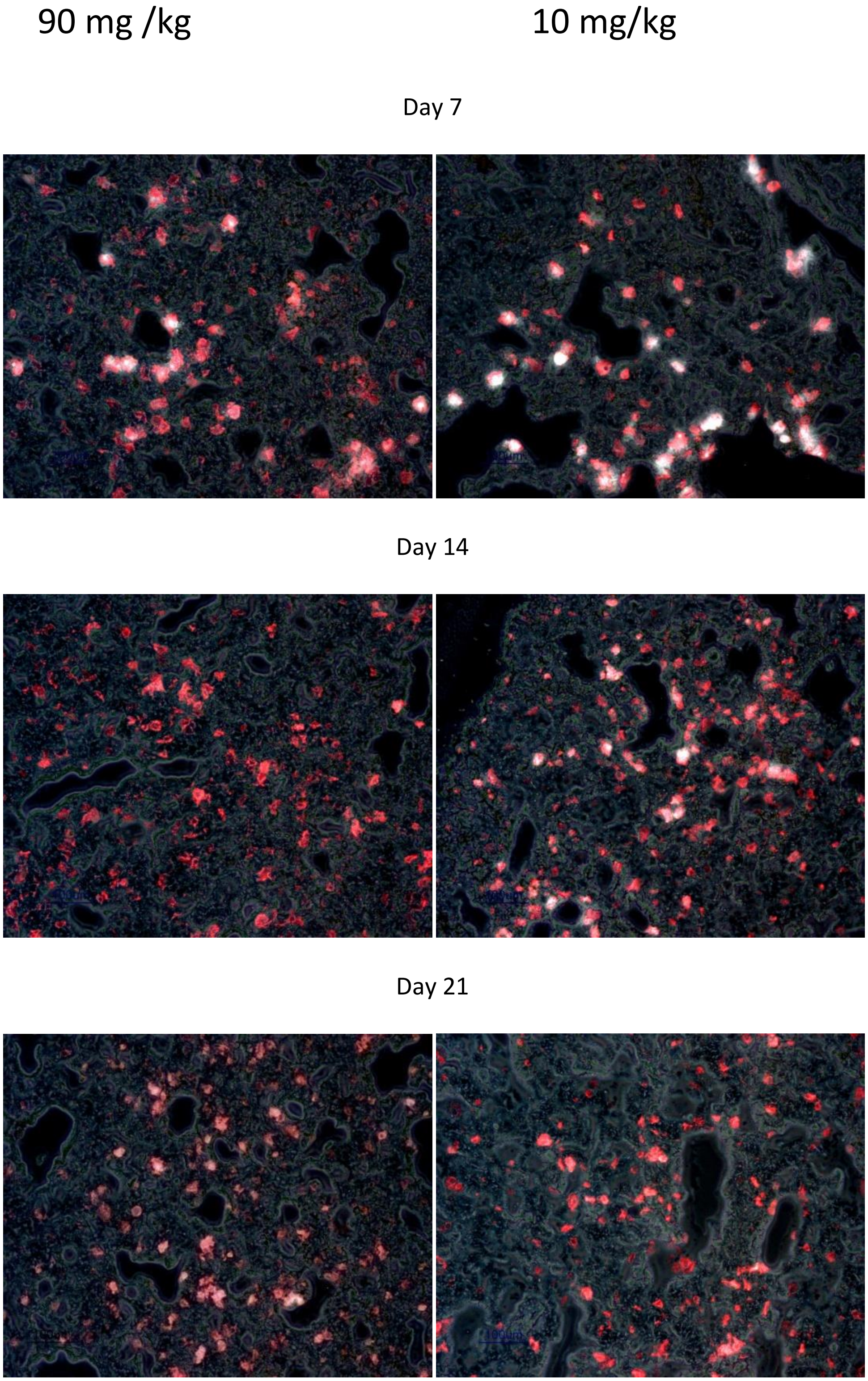

Figure S1-3 . Fluorescence of amikacin-TAMRA (white) and $DiIC_{18}$(5)DS (red) and their co-localization (pink) in lung tissues after 27 daily doses of 90 mg/kg (left) or 10 mg/kg (right) of LAI followed by a single dose of 90 mg/kg of dual-labeled LAI *(day 28).* $DiIC_{18}$(5)-DS 1′-Dioctadecyl-3,3,3′,3′-tetramethylindodicarbocyanine-5,5′-disulfonic acid; LAI, liposomal amikacin for inhalation; TAMRA, tetramethylrhodamine.

90 mg /kg 10 mg/kg

Day 28

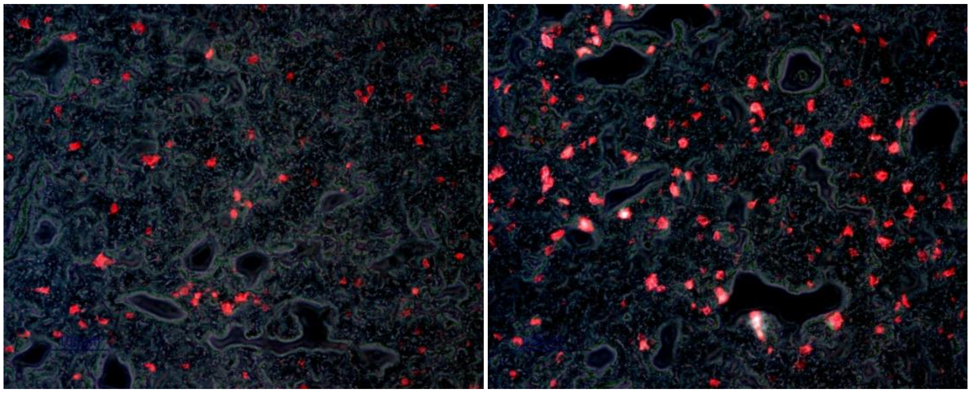

Figure S2. Panoramic images of rat caudal lung post inhalation of 27 daily doses of 90 mg /kg of LAI followed by a single dose of 90 mg /kg of dual-labeled LAI [4-hr post Inhalation (A), day 3 post Inhalation (B), day 28 post Inhalation]. DiIC$_{18}$(5)-DS 1′-Dioctadecyl-3,3,3′,3′-tetramethylindodicarbocyanine-5,5′-disulfonic acid; LAI, liposomal amikacin for inhalation; TAMRA, tetramethylrhodamine.

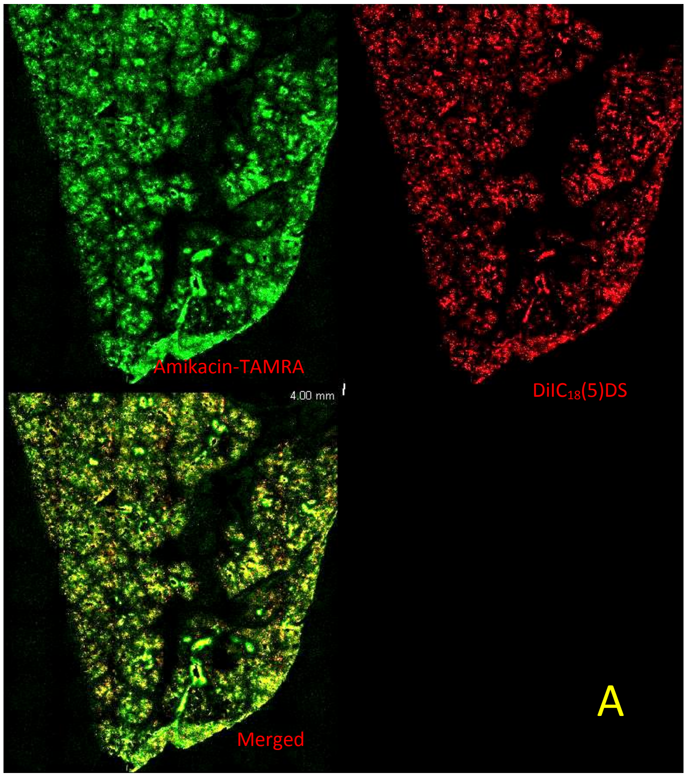

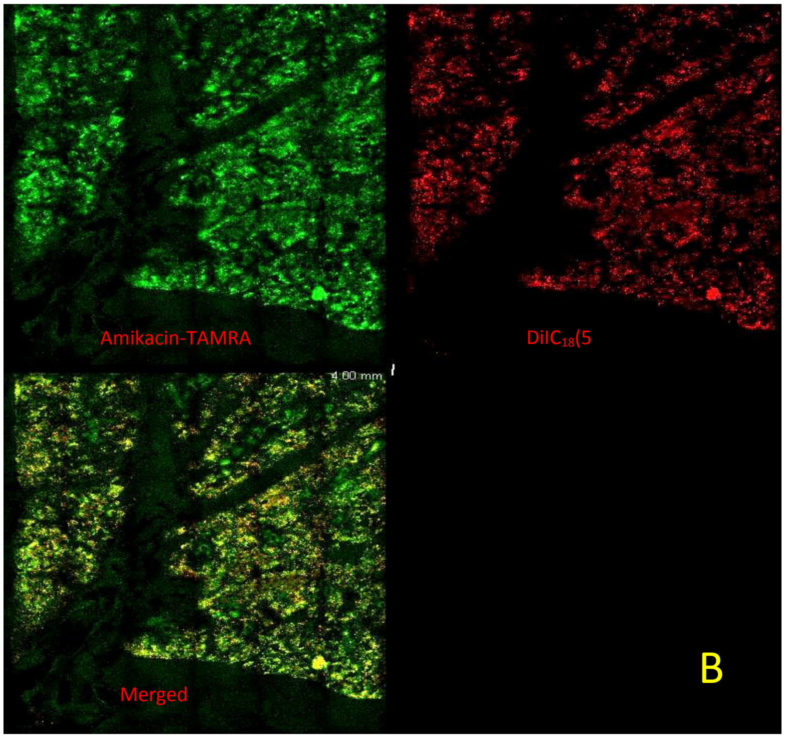

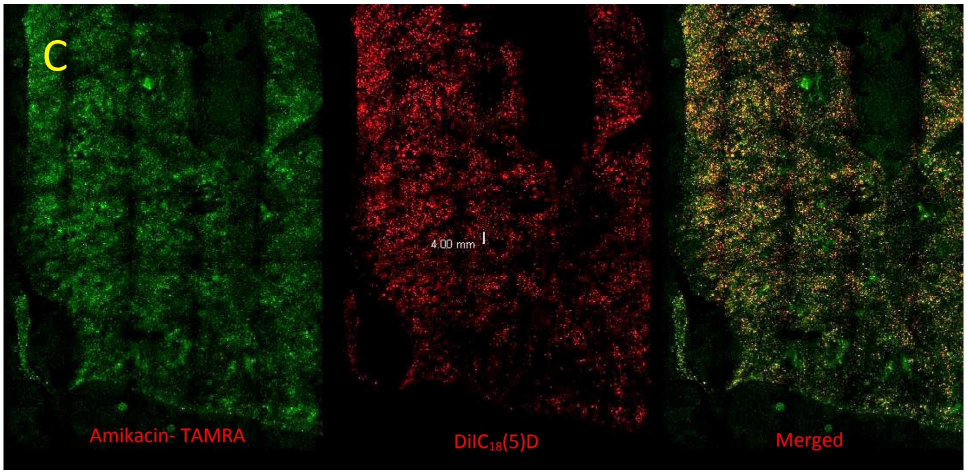

Images were acquired using a Leica SP5 Spectral confocal microscope with a motorized stage (Leica, Germany). Images were collected with a 10X Plan Apochromat 0.4 N A objective at a scan speed of 400 Hz and a confocal pin hole set of 2 airy units. Optical slices were collected at 3-µm intervals (12-18 slices per field) and tissue sections were imaged in tile scan mode. Sections were projected using maxim mode projection and then the fields were stitched together to form full-field images. The green channel was assigned to fluorescence excitation at 514 nm and emission between 520 and 580 nm. The red channel was assigned to fluorescence excitation at 633 nm and emission from 650 to 750 nm. The corresponding images from the red and green channels were overlaid electronically to detect co-localization of both fluorescent probes. The bronchus enters the lung form the top right side on Figure S2 A and C, and enters the lung on the lower left side on Figure S2 C.

Figure S3 . Fluorescence of amikacin-TAMRA (white) and $DiIC_{18}(5)DS$ (red) and their co-localization (pink) in BAL cells after 27 daily doses of 10 mg/kg (left) or 90 mg/kg (right) of LAI followed by a single dose of 90 mg/kg of dual-labeled LAI [immediately post dose (A), day 14 (B), day 28 (C)]. $DiIC_{18}(5)$-DS 1′-Dioctadecyl-3,3,3′,3′-tetramethylindodicarbocyanine-5,5′-disulfonic acid; LAI, liposomal amikacin for inhalation; TAMRA, tetramethylrhodamine.

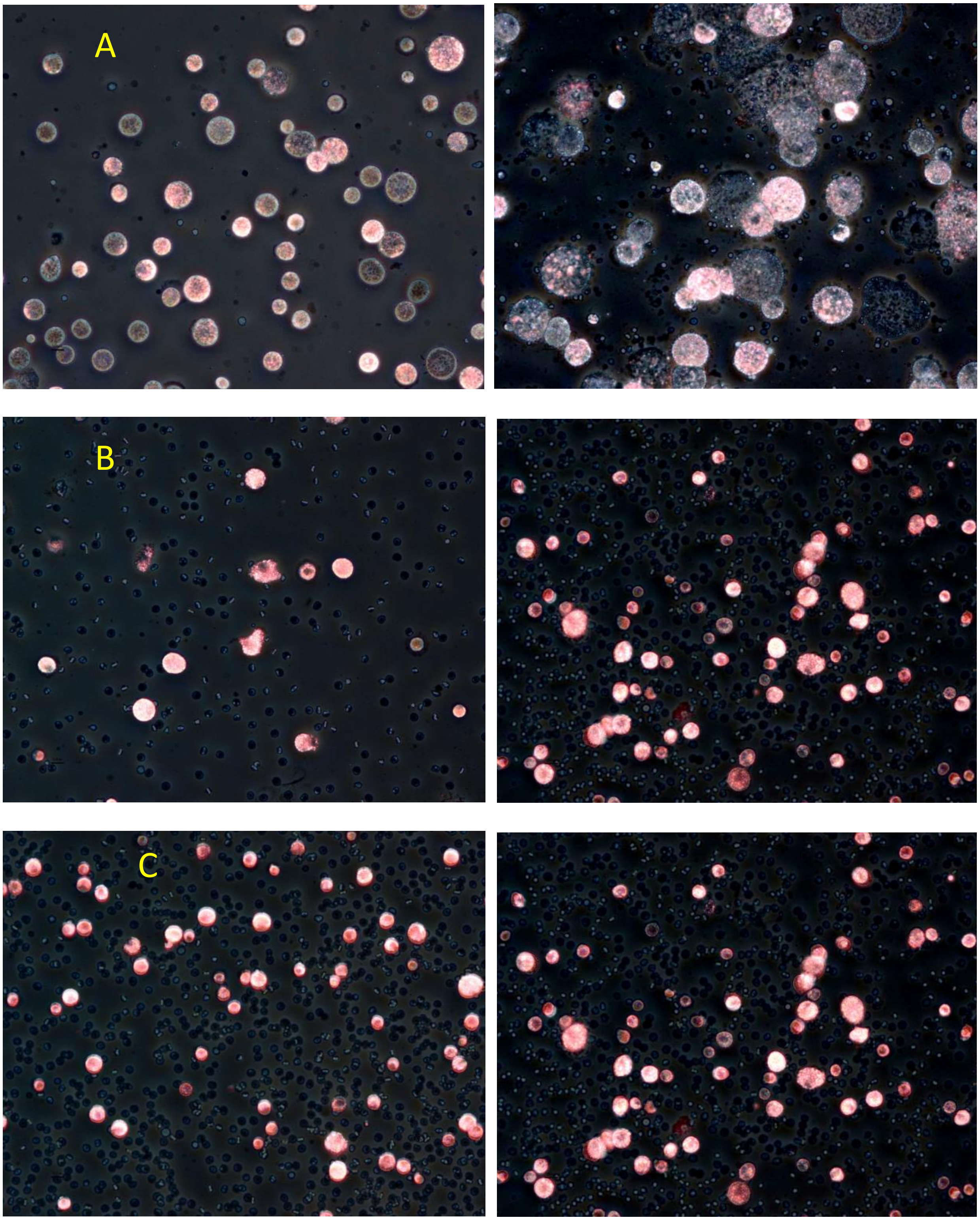

Supplement: Supplemental material [file AAC.00700-16_zac011165625so1.pdf]
